# Supplementary material for: Fragmentation and correlations in a rotating Bose–Einstein condensate undergoing breakup
Source: Sci Rep. 2023 Feb 27;13:3343. doi: 10.1038/s41598-023-29516-w (PMC9971194; doi:10.1038/s41598-023-29516-w)
Supplement: Supplementary file 1 — Supplementary Information. [file 41598_2023_29516_MOESM1_ESM.pdf]

Supplemental material to fragmentation and correlations in a rotating Bose-Einstein condensate undergoing breakup

Sunayana Dutta,[1,2,*] Axel U. J. Lode,[3] and Ofir E. Alon[1,2]

[1]*Department of Physics, University of Haifa, Haifa 3498838, Israel*

[2]*Haifa Research Center for Theoretical Physics and Astrophysics, University of Haifa, Haifa 3498838, Israel*

[3]*Institute of Physics, Albert-Ludwig University of Freiburg, Hermann-Herder-Strasse 3, 79104 Freiburg, Germany*

(Dated: February 1, 2023)

* sdutta@campus.haifa.ac.il

This Supplemental material provides further many-body analysis that supports our main results. Sec. S1 benchmarks the multiconfigurational time-dependent Hartree for bosons (MCTDHB) method [1–3] in a harmonic interaction model (HIM) under rotation that gives an exactly solvable many-body model. This method is employed for the numerical computations of the main results. Sec. S2 gives the convergence checks of the ground state energy per particle of our main results with respect to the number of self-consistent orbitals in different anharmonic traps, namely, elongated, three-fold symmetric, and four-fold symmetric traps. Sec. S3 illustrates the convergence of natural occupations in these confining traps with respect to the number of orbitals. Sec. S4 presents the many-particle variances discussed in the main text, along with their convergences with respect to the number of self-consistent orbitals. This section is further divided into two subsections. The first subsection deals with convergence of the position and momentum variances. Convergence with respect to the number of self-consistent orbitals of the expectation value of the angular momenta and angular momentum variance is elaborated in the second subsection that supports our main results. Sec. S5 comprises the structure of the natural orbitals for slow, fast and faster rotaions. Finally, Sec. S6 concludes the quantum features in the resonance regime.

S1. BENCHMARKING OF MCTDHB IN THE ROTATING FRAME: AN EXACTLY SOLVABLE MANY-BODY MODEL

In this section, we present a numerical benchmark of the MCTDHB method in the rotating frame in two dimensions 2D. The many-body Hamiltonian in the rotating frame can be written as

$$\hat{H}(\mathbf{r_1}, \mathbf{r_2}, ..., \mathbf{r_N}) = \sum_{j=1}^{N} \hat{h}(\mathbf{r}_i) + \sum_{j<k}^{N} \hat{W}(\mathbf{r}_j, \mathbf{r}_k) - \omega_r \hat{L}_Z, \quad \text{(S1)}$$

where the single-particle Hamiltonian is $\hat{h}(\mathbf{r}) = -\frac{1}{2}\frac{\partial^2}{\partial \mathbf{r}^2} + \hat{V}(\mathbf{r})$. We consider a harmonic interaction model (HIM) in 2D. In this HIM, the confining trapping potential $\hat{V}(\mathbf{r})$ is a translated 2D harmonic oscillator of the following form

$$\hat{V}(\mathbf{r}) = \frac{1}{2} m\omega^2 [(x-L)^2 + y^2], \quad \text{(S2)}$$

and the two-body interaction potential $\hat{W}(\mathbf{r}_j, \mathbf{r}_k)$ is also harmonic

$$\hat{W}(\mathbf{r}_j, \mathbf{r}_k) = \lambda_0 (\mathbf{r}_j - \mathbf{r}_k)^2, \quad \text{(S3)}$$

where the two-body interaction strength λ_0 defines the mean-field interaction parameter as $\Lambda = \lambda_0(N-1)$, ω_r is the rotation frequency, and $\hat{L}_Z = \sum_{j=1}^{N} \hat{l}_{z_j} = \sum_{j=1}^{N} \left(\hat{x}_j \hat{p}_{y_j} - \hat{y}_j \hat{p}_{x_j}\right)$ is the many-body angular momentum operator.

Now, the ground state solution of the HIM in the rotating frame is obtained by solving the many-boson Schrödinger equation given as

$$\hat{H}(\mathbf{r})\Psi(\mathbf{r}) = E\Psi(\mathbf{r}). \tag{S4}$$

The N-boson wave function of the ground state in the rotating frame is obtained as

$$\Psi(\mathbf{r}_1, \mathbf{r}_2, ..., \mathbf{r}_N) = \left(\frac{m\Omega}{\pi}\right)^{\frac{N-1}{4}} \left(\frac{m\omega}{\pi}\right)^{\frac{1}{4}} e^{+i\beta\sum_{j=1}^{N} y_j} e^{-\frac{1}{2}m\omega\sum_{j=1}^{N}(x_j - x_0)^2} e^{-\frac{1}{2}m\omega\sum_{j=1}^{N} y_j^2}, \tag{S5}$$

where the dressed frequency is

$$\Omega = \sqrt{\omega^2 + \frac{2\lambda_0 N}{m}},$$

and the translation and momentum parameters are

$$x_0 = \frac{1}{1 - (\frac{\omega_r}{\omega})^2} L \tag{S6}$$

and

$$\beta = m\omega_r x_0 = m\omega_r \frac{1}{1 - (\frac{\omega_r}{\omega})^2} L. \tag{S7}$$

Thus, we can evaluate the ground state properties, such as, the energy, densities, and reduced density matrices from the many-boson wavefunction Ψ in Equation (S5).

As we know, the ground state energy per particle in the HIM without rotation is given as [4]

$$\frac{E(\omega_r = 0)}{N} = \left[(N-1)\Omega + \omega\right] = \left[(N-1)\sqrt{\omega^2 + \frac{2\lambda_0 N}{m}} + \omega\right]. \tag{S8}$$

Finally, in the rotating frame the energy per particle is obtained as

$$\frac{E(\omega_r)}{N} = \frac{E(\omega_r = 0)}{N} - \frac{1}{2} m\omega^2 \frac{(\frac{\omega_r}{\omega})^2}{1 - (\frac{\omega_r}{\omega})^2} L^2. \tag{S9}$$

Now we are going to evaluate the expectation values and variances of many-particle observables, explicitly the position, momentum, and angular momentum operators. The expectation values of the many-particle position operators are given as

$$\frac{\langle \hat{X} \rangle}{N} = x_0 = \frac{1}{1 - (\frac{\omega_r}{\omega})^2} L, \qquad \frac{\langle \hat{Y} \rangle}{N} = 0, \tag{S10}$$

and the expectation values of the many-particle momentum operators are given as

$$\frac{\langle \hat{P}_X \rangle}{N} = 0, \qquad \frac{\langle \hat{P}_Y \rangle}{N} = \beta = m\omega_r x_0 = m\omega_r \frac{1}{1-(\frac{\omega_r}{\omega})^2} L. \tag{S11}$$

Similarly, the expectation value of the many-particle angular momentum operator reads

$$\frac{\langle \hat{L}_Z \rangle}{N} = x_0 \beta = m\omega_r \left[\frac{L}{1-(\frac{\omega_r}{\omega})^2} \right]^2. \tag{S12}$$

The many-particle position variances per particle are obtained as,

$$\frac{1}{N}\Delta^2_{\hat{X}} = \frac{1}{N}\Delta^2_{\hat{Y}} = \frac{1}{2m\omega}, \tag{S13}$$

and the many-particle momentum variances per particle are the following

$$\frac{1}{N}\Delta^2_{\hat{P}_X} = \frac{1}{N}\Delta^2_{\hat{P}_Y} = \frac{m\omega}{2}. \tag{S14}$$

It is evident that the many-particle variances of the position and momentum operators in the rotating frame boil down to those for the non-rotating case.

However, the many-particle angular momentum variance per particle is affected by the rotation and takes on the following form

$$\frac{1}{N}\Delta^2_{\hat{L}_Z} = x_0^2 \frac{1}{N}\Delta^2_{\hat{P}_Y} + \beta^2 \frac{1}{N}\Delta^2_{\hat{X}} = \frac{1}{2} m\omega \frac{\left[1+(\frac{\omega_r}{\omega})^2\right]}{\left[1-(\frac{\omega_r}{\omega})^2\right]^2} L^2. \tag{S15}$$

With a solvable many-boson model in the rotating frame, we can now proceed and benchmark MCTDHB in the rotating frame. In the following simulations, we work in the units $\hbar = m = \omega = 1$. To compute the ground state of MCTDHB in the rotating frame, we consider $N = 10$ weakly interacting bosons with repulsive interaction parameter $\Lambda = -0.1$. The box extensions of the confining potential are chosen to be $= [\mathbf{-12}, \mathbf{12}) \times [\mathbf{-12}, \mathbf{12})$ with $\mathbf{128} \times \mathbf{128}$ DVR functions to represent each of the orbitals. We translated the confining potential by $L = 2$ in Equation (S2).

In Table S1, we show the numerical convergence of the energy per particle $\frac{E}{N}$. Exact analytical versus numerical MCTDHB ground state energies are obtained for three different rotation frequencies ω_r. The numerical convergence of the many-body energy per particle is achieved by using $M = 6$ self-consistent orbitals. Thus, we conclude that for all ω_r, the ground state energy of the system is converged.

ω_r	$\frac{E}{N}_{numerical}$	$\frac{E}{N}_{analytical}$
0	0.89372	0.89372
0.5	0.22705	0.22705
0.8	-2.66183	-2.66183

TABLE S1. Benchmarking of the ground state many-body energy E/N with respect to the rotation frequency ω_r. $M = 6$ self-consistent orbitals are used. The ground state energies shown are in dimensionless units.

ω_r	$\frac{1}{N}\langle\hat{X}\rangle_{numerical}$	$\frac{1}{N}\langle\hat{X}\rangle_{analytical}$	$\frac{1}{N}\langle\hat{Y}\rangle_{numerical}$	$\frac{1}{N}\langle\hat{Y}\rangle_{analytical}$
0	2.00000	2.00000	0.00000	0.00000
0.5	2.66666	2.66666	0.00000	0.00000
0.8	5.55555	5.55555	0.00000	0.00000
ω_r	$\frac{1}{N}\Delta^2_{\hat{X}numerical}$	$\frac{1}{N}\Delta^2_{\hat{X}analytical}$	$\frac{1}{N}\Delta^2_{\hat{Y}numerical}$	$\frac{1}{N}\Delta^2_{\hat{Y}analytical}$
0	0.50000	0.50000	0.50000	0.50000
0.5	0.50000	0.50000	0.50000	0.50000
0.8	0.50000	0.50000	0.50000	0.50000

TABLE S2. Benchmarking of the expectation values and variances of the many-particle position operators along the x- and y- directions with respect to the rotation frequency ω_r computed with $M = 6$ self-consistent orbitals. All the quantities are shown in dimensionless units.

Table S2 shows the comparison of the exact analytical and numerical MCTDHB results of the many-particle position variances for various rotation frequencies ω_r. The numerical convergence of MCTDHB results computed with $M = 6$ self-consistent orbitals are clearly evident from the table.

Table S3 and S4 correspond to benchmarking of the many-particle momentum and angular momentum variances for various rotation frequencies computed with $M = 6$ self-consistent orbitals. As the many-particle variances of the momentum and angular momentum are very sensitive quantities, it is generally more difficult to achieve numerical convergence of the analytical and numerical results. However, from these tables, it is evident that the MCTDHB results are converged with exact analytical results.

ω_r	$\frac{1}{N}\langle\hat{P}_X\rangle_{numerical}$	$\frac{1}{N}\langle\hat{P}_X\rangle_{analytical}$	$\frac{1}{N}\langle\hat{P}_Y\rangle_{numerical}$	$\frac{1}{N}\langle\hat{P}_Y\rangle_{analytical}$
0	0.00000	0.00000	0.00000	0.00000
0.5	0.00000	0.00000	1.33333	1.33333
0.8	0.00000	0.00000	4.44444	4.44444
ω_r	$\frac{1}{N}\Delta^2_{\hat{P}_X\,numerical}$	$\frac{1}{N}\Delta^2_{\hat{P}_X\,analytical}$	$\frac{1}{N}\Delta^2_{\hat{P}_Y\,numerical}$	$\frac{1}{N}\Delta^2_{\hat{P}_Y\,analytical}$
0	0.50000	0.50000	0.50000	0.50000
0.5	0.50000	0.50000	0.50000	0.50000
0.8	0.50000	0.50000	0.50000	0.50000

TABLE S3. Benchmarking of the expectation values and variances of the many-particle momentum operators along the x- and y- directions with respect to the rotation frequency ω_r obtained with $M = 6$ self-consistent orbitals. All the quantities are shown in dimensionless units.

ω_r	$\frac{1}{N}\langle\hat{L}_Z\rangle_{numerical}$	$\frac{1}{N}\langle\hat{L}_Z\rangle_{analytical}$	$\frac{1}{N}\Delta^2_{\hat{L}_Z\,numerical}$	$\frac{1}{N}\Delta^2_{\hat{L}_Z\,analytical}$
0	0.00000	0.00000	2.00000	2.00000
0.5	3.55555	3.55555	4.44444	4.44444
0.8	24.6913	24.6913	25.3086	25.3086

TABLE S4. Benchmarking of the expectation values and variances of the many-particle angular momentum operator with respect to the rotation frequency ω_r computed with $M = 6$ self-consistent orbitals. All the quantities are shown in dimensionless units.

S2. CONVERGENCE OF THE GROUND STATE ENERGY

In this section, we investigate and report the convergence of the many-particle ground state energy $\frac{E}{N}$ for the three confining potentials, namely the elongated trap

$$V(\mathbf{r}) = \frac{1}{4}(0.8x^2 + y^2)^2, \tag{S16}$$

the three-fold symmetric trap

$$V(\mathbf{r}) = \frac{1}{5}(x^2 + y^2)^2 + \frac{1}{5}(x^2 y - \frac{1}{3}y^3), \tag{S17}$$

and, finally, the four-fold symmetric trap defined as

$$V(\mathbf{r}) = \frac{1}{4}(x^4 + y^4). \tag{S18}$$

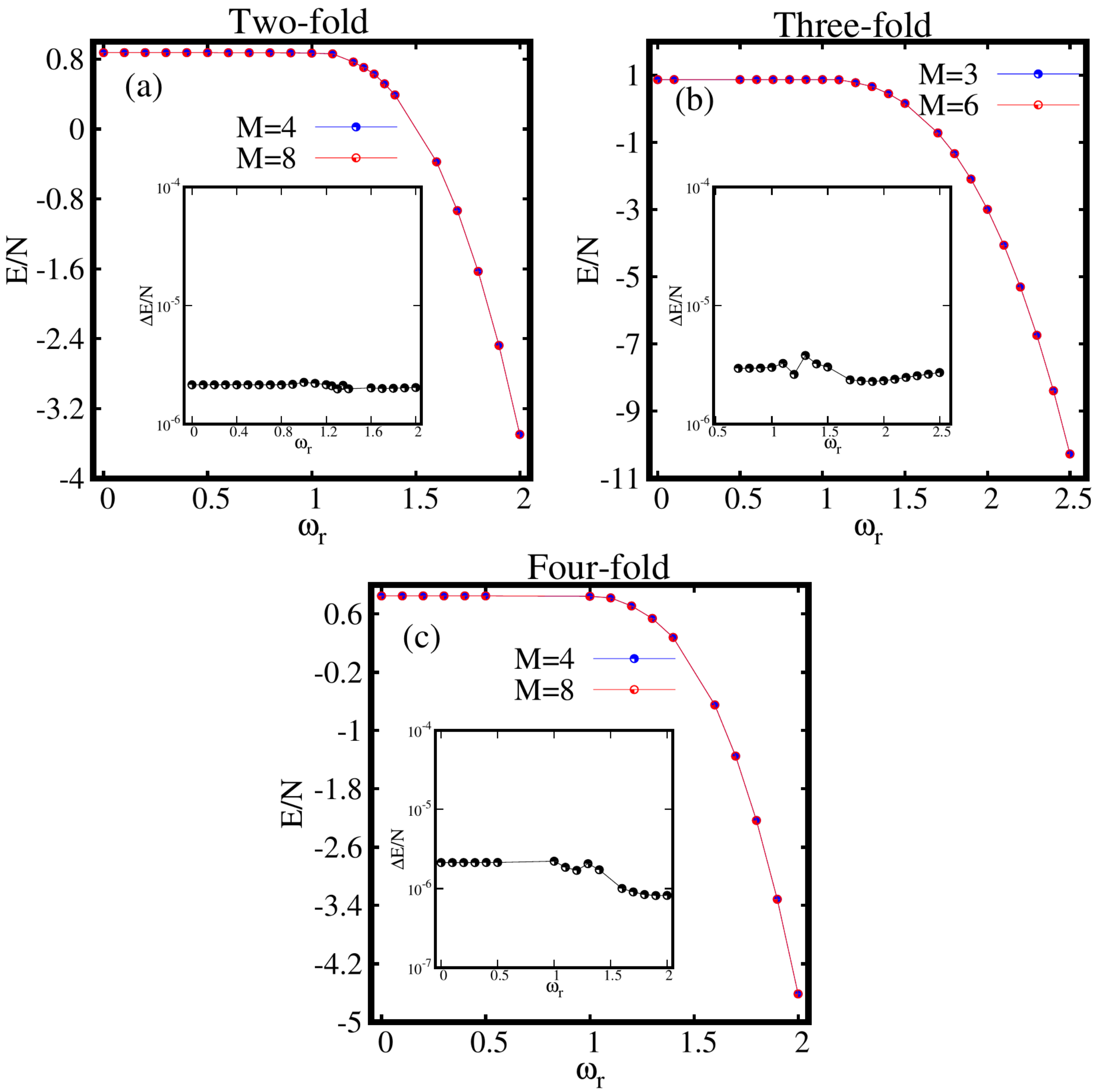

FIG. S1. Many-body ground state energy per particle $\frac{E}{N}$ as a function of the rotation frequency ω_r. (a) for the elongated trap $M = 8$ self-consistent orbitals are used to show the convergence of the $M = 4$ results, (b) for the three-fold symmetric trap $M = 6$ self-consistent orbitals are used to check the convergence of $M = 3$ results, and finally in (c) $M = 8$ self-consistent orbitals are used to check the convergence of $M = 4$ results of the four-fold symmetric trap. The inset shows the energy difference per particle as defined in Equation (S19). The quantities shown are dimensionless.

The convergence of the many-body energies reported in the main text with M self-consistent orbitals is here verified by taking $2M$ self-consistent orbitals and comparing the respective results.

Figure S1 shows the behavior of the ground state energy $\frac{E}{N}$ as a function of the rotation frequency ω_r for different numbers of self-consistent orbitals for three confining potentials given by Equations (S16), (S17), and (S18). The inset in all the three panels of Figure S1 display the energy difference between the energies computed for two different orbital numbers M,

$$\frac{\Delta E}{N} = \frac{E_M}{N} - \frac{E_{2M}}{N}. \tag{S19}$$

Figure S1(a) corresponds to the elongated trap [Equation (S16)]. The convergence of energy is checked for two different orbitals $M = 4, 8$. We find that the two energy curves $\frac{E}{N}$ computed with $M = 4, 8$ orbitals fall on top of each other for all the frequencies. It is evident that our results converges with respect to the number of orbitals for elongated trap. Similar convergence is observed in case of the three-fold symmetric trap with $M = 3, 6$ orbitals in Figure S1(b) [see Equation (S17)] and the four-fold symmetric trap [Equation (S18)] with $M = 4, 8$ orbitals as evident from Figure S1(c). We conclude that the ground state energies are fully converged with respect to the number of orbitals in all the three confining potentials at all rotation frequencies.

S3. CONVERGENCE OF THE NATURAL OCCUPATIONS

The behavior of the natural occupations n_j/N for the confining potentials defined in Equations (S16), (S17), and (S18), as a function of the rotation frequency ω_r are shown in Figures S2 for different orbital numbers M. It is observed that all the natural occupations are fully converged for the three confining potentials. The inset in Figure S2(b) shows the variation of the smallest natural occupations $\frac{n_4}{N}$, $\frac{n_5}{N}$, and $\frac{n_6}{N}$ with the rotation frequency ω_r for the three-fold symmetric trap. All the three natural occupations stays minimal with $\frac{n_4}{N} \sim \frac{n_5}{N} \sim \frac{n_6}{N} \sim 10^{-6}$ for all ω_r. Similarly, the inset of Figure S2(c) depicts the natural occupations $\frac{n_5}{N} \sim 10^{-6}$, $\frac{n_6}{N} \sim \frac{n_7}{N} \sim \frac{n_8}{N} \sim 10^{-7} - 10^{-6}$ for the four-fold symmetric trap. The convergence of the natural occupations is evident in all three confining traps.

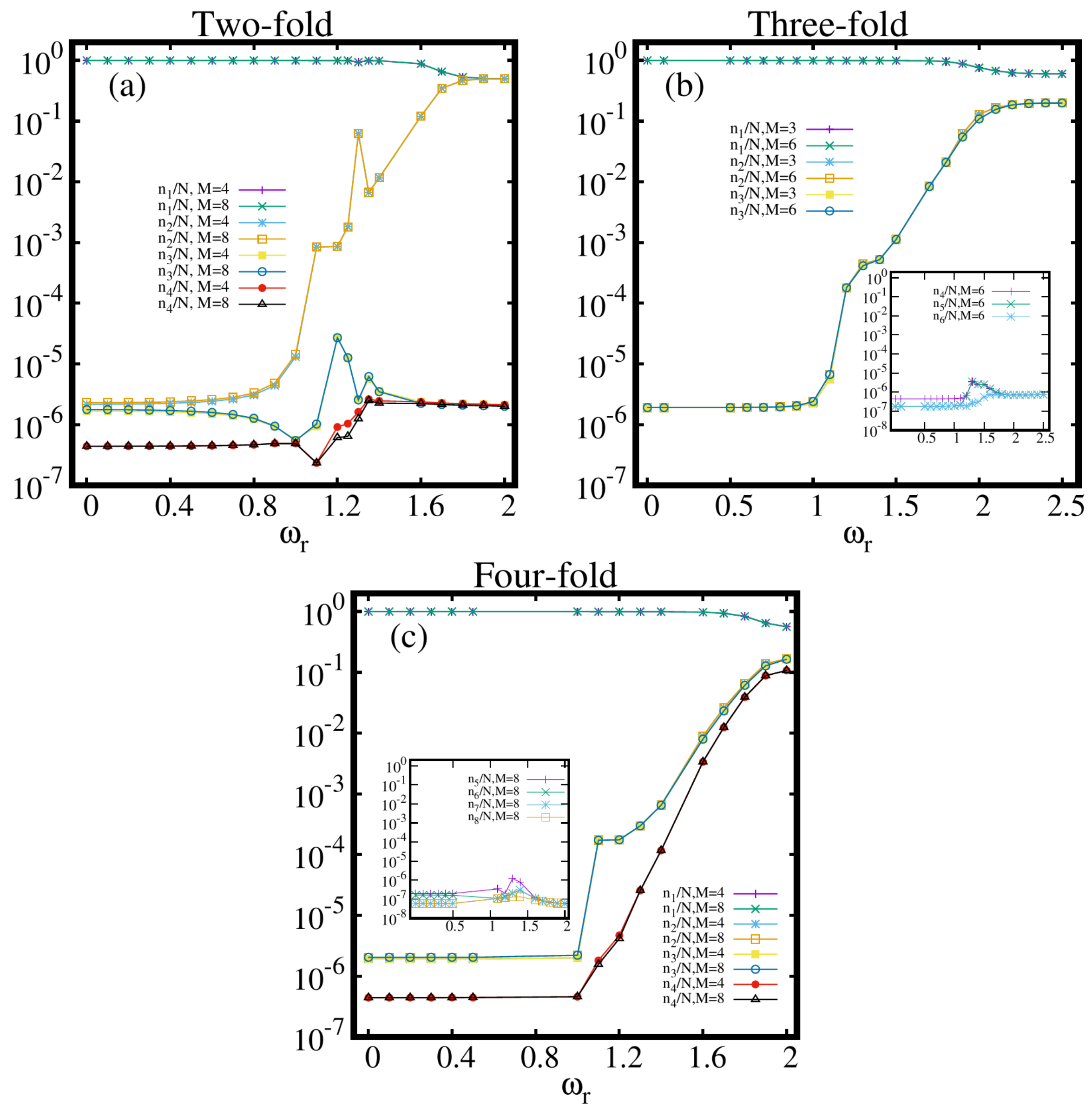

FIG. S2. Convergence of the natural occupations with respect to the number of natural orbitals. (a) for the elongated trap with M=4,8 self-consistent orbitals, (b) for the three-fold symmetric trap with M=3,6 self-consistent orbitals, and (c) for the four-fold symmetric trap with M=4,8 self-consistent orbitals. The inset in panels (b) and (c) depict the variation of the smallest natural occupations with rotation.

S4. CONVERGENCE OF THE POSITION, MOMENTUM, AND ANGULAR MOMENTUM VARIANCES

The variance of a many-particle operator $\hat{A} = \sum_{j=1}^{N} \hat{a}_j$ per particle is defined as

$$\frac{1}{N}\Delta^2_{\hat{A}} = \frac{1}{N}\left[\langle\Psi|\hat{A}^2|\Psi\rangle - \langle\Psi|\hat{A}|\Psi\rangle^2\right]. \tag{S20}$$

The detail discussion is included in the main text. Here, we report the convergence of many-particle variances of the position, momentum and angular momentum operators [5, 6] for all the three confining potentials in the rotating frame with respect to the number of orbitals.

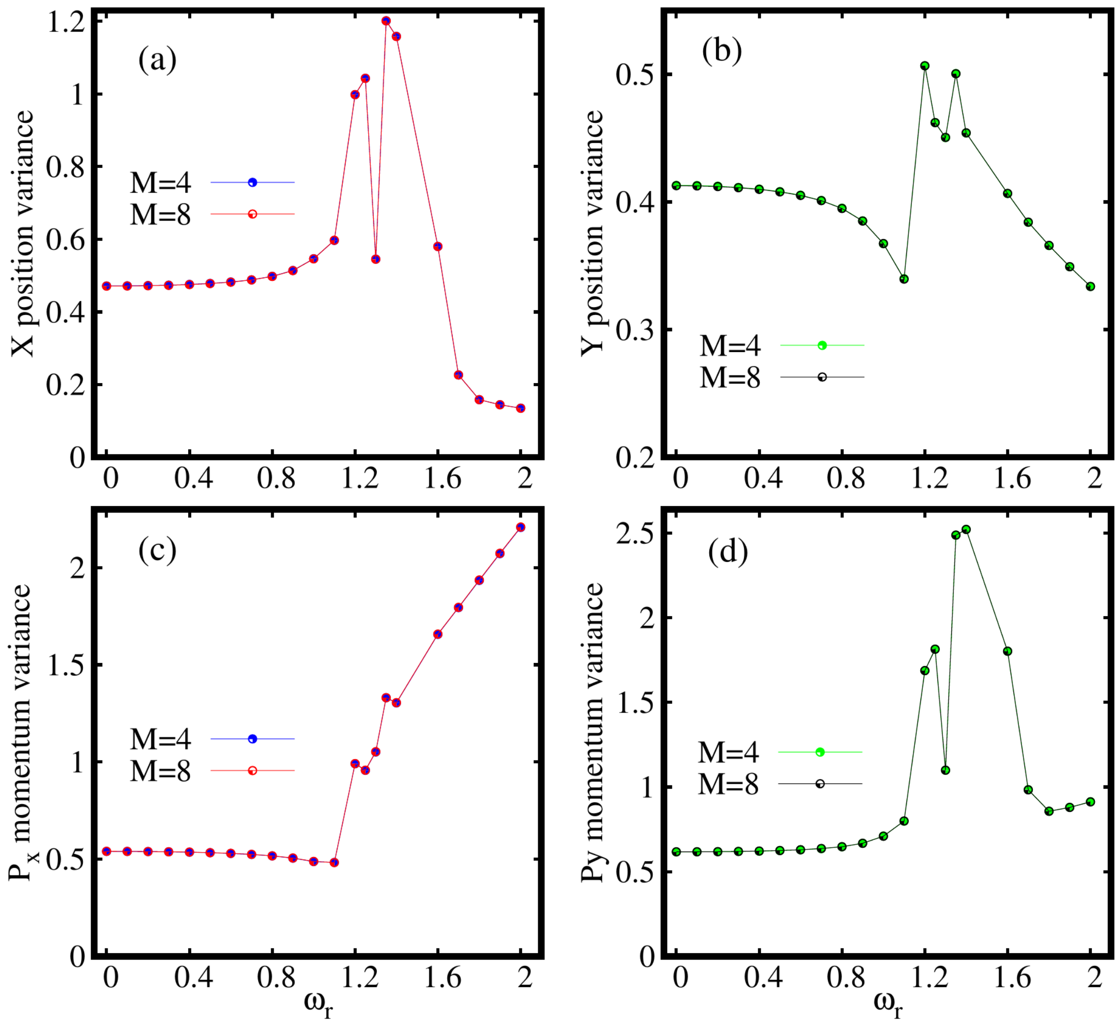

FIG. S3. Convergence of the many-particle position variances (a)-(b) and momentum variances (c)-(d) computed for the elongated trap with M=4,8 self-consistent orbitals along the x- and y-directions.

A. The position and momentum variances

This section presents the convergence of many-particle position variances $\frac{1}{N}\Delta^2_{\hat{X}}$, $\frac{1}{N}\Delta^2_{\hat{Y}}$ and many-particle momentum variances $\frac{1}{N}\Delta^2_{\hat{P}_X}$, $\frac{1}{N}\Delta^2_{\hat{P}_Y}$ with respect to the number of orbitals M for the three confining potentials.

Figures S3(a)-(b) show the convergence of the many-particle position variances and Figures S3(c)-(d) correspond to the many-particle momentum variances for the elongated trap [Equation (S16)] with $M = 4, 8$ orbitals.

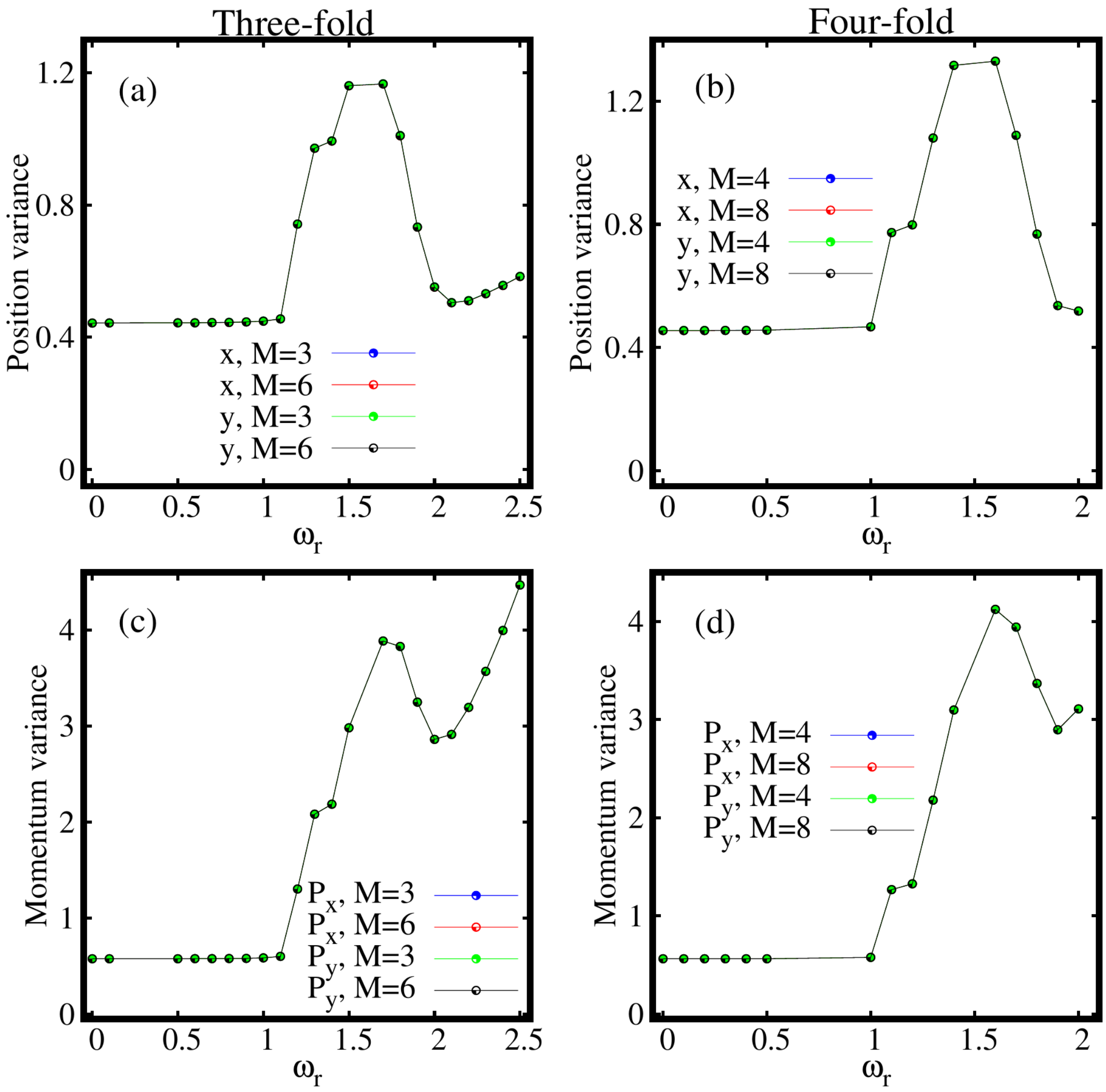

FIG. S4. Convergence of the many-particle position (a) and momentum variances (c) with M=3,6 self-consistent orbitals for the three-fold symmetric trap. Panels (b) and (d) correspond to many-particle position and momentum variances with M=4,8 self-consistent orbitals for the four-fold symmetric trap.

Figures S4(a),(c) correspond to the many-particle position and momentum variances for three-fold symmetric trap [Equation (S17)]. Similarly, Figures S4(b),(d) depict the many-particle position and momentum variances for the four-fold symmetric trap [Equation (S18)].

It is evident that the convergence with respect to the number orbitals of the many-particle variances of the position and momentum operators for all the three confining potentials is excellent.

B. Angular momentum properties

Before we discuss the angular momentum variance, we wish to show that the expectation value of the angular momentum operator discussed in the main text converges at the many-body level. Then, we proceed to the angular momentum variance, and augment the text by its analysis.

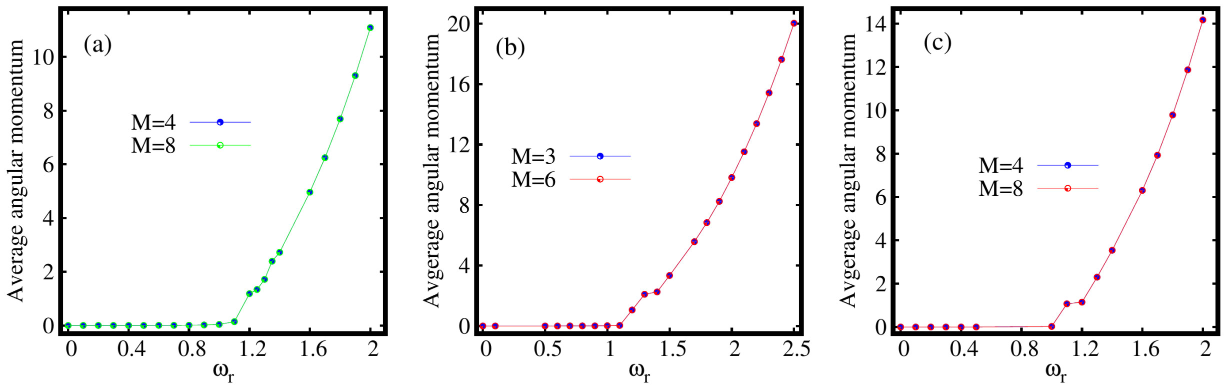

FIG. S5. Convergence of the expectation values of the angular momentum operator per particle. (a) for the elongated trap with M=4,8 self-consistent orbitals, (b) for the three-fold symmetric trap with M=3,6 self-consistent orbitals and (c) for the four-fold symmetric trap with M=4,8 self-consistent orbitals.

Figure (S5) proves the convergence of the expectation values of angular momentum operator $\hat{L}_Z = \sum_{j=1}^{N} \hat{l}_{z_j}$ per particle $\frac{1}{N}\langle\Psi|\hat{L}_Z|\Psi\rangle$, with respect to the number of orbitals M for the three confining potentials; the elongated trap [Equation (S16)], the three-fold symmetric trap [Equation (S17)] and the four-fold symmetric trap [Equation (S18)].

Finally, we discuss the convergence of the many-particle angular momentum variance $\frac{1}{N}\Delta^2_{\hat{L}_Z}$ in all the three confining traps. It is evident from Figure (S6) that the angular momentum variances are fully converged with respect to the number of orbitals for all the

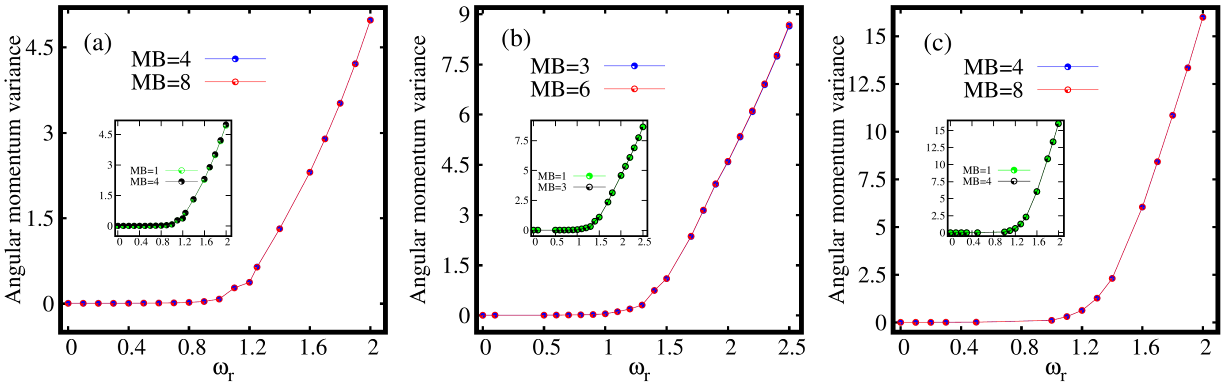

FIG. S6. Convergence of the angular momentum variance. (a) for the elongated trap with M=4,8 self-consistent orbitals, (b) for the three-fold symmetric trap with M=3,6 self-consistent orbitals and (c) for the four-fold symmetric trap with M=4,8 self-consistent orbitals. The insets of (a), (b), and (c) demonstrate the angular momentum variances at the mean-field and many-body levels that are essentially the same in our rotating traps.

three confining potentials at all ω_r. The insets of Figure (S6) demonstrate that in the present rotating traps and interaction strengths the mean-field and many-body angular momentum variances essentially coincide.

S5. STRUCTURE OF THE NATURAL ORBITALS FOR SLOW, FAST AND FASTER ROTATIONS

As we already know, the MCTDHB calculation is many-body in nature and is represented by the time-dependent permanents. To quantify various quantum properties, such as, correlations and coherence, reduced density matrices, RDM and their eigenvalues are used in MCTDHB. The RDM is diagonalized with natural orbitals as,

$$\frac{\rho^{(1)}(\mathbf{r},\mathbf{r}')}{N} = \sum_j n_j \phi_j^{(NO),*}(\mathbf{r}')\phi_j^{(NO)}(\mathbf{r}). \tag{S21}$$

The eigenvalues, n_j and eigenvectors, $\phi_j^{(NO)}$ are termed as the natural occupations and natural orbitals respectively. For single orbital, the MCTDHB ansatz becomes identical to the wavefunction ansatz of the time-dependent Gross–Pitaevskii (TDGP) theory. However, several natural orbitals are required in the many-body calculation. For example, in the elongated trap, four natural orbitals are used for numerical calculations. MCTDHB wavefunction with rotation is complex in nature. Figure (S7) presents the variation of the real

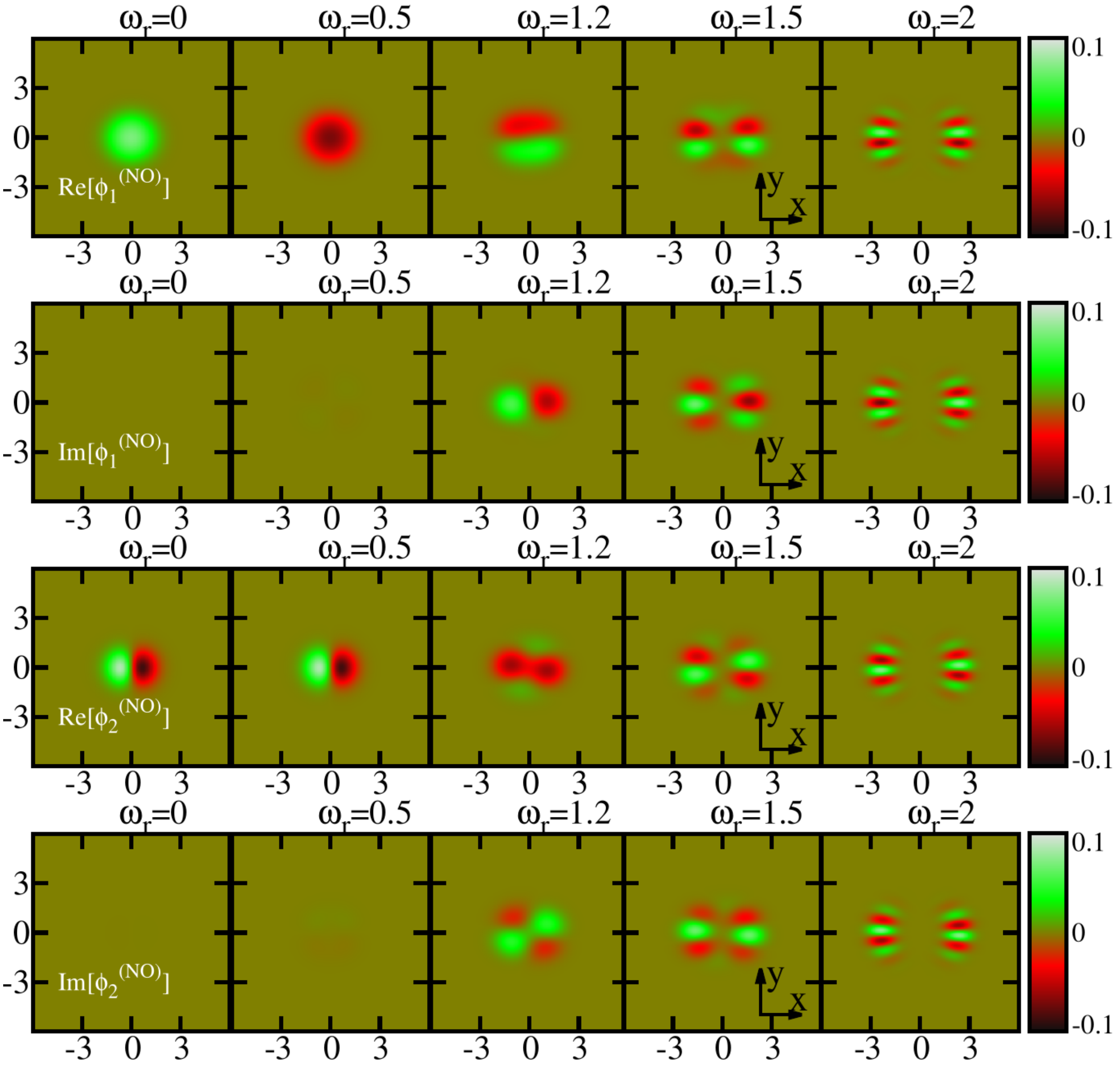

FIG. S7. Variation of the real and imaginary components of the two leading natural orbitals, ϕ_1, ϕ_2 with respect to different rotation frequencies, ω_r for elongated trap.

and imaginary components of the two leading natural orbitals, ϕ_1 and ϕ_2 for slow, fast and faster rotations in the elongated trap computed at the many-body level. It is observed that without rotation ($\omega_r = 0$), the first natural orbital, ϕ_1 has a single maximum centered at the origin of the trap. The second natural orbital, ϕ_2 has a single nodal point. Both the natural orbitals are completely real without rotation as ϕ_1, ϕ_2 don't have any imaginary components. These features persist even for slow rotation as evident from Figure (S7). For fast rotations ($\omega_r \sim 1.2$), ϕ_1 and ϕ_2 become complex with the development of the imaginary components both in ϕ_1 and ϕ_2. This signifies the onset of some phases in the system. For

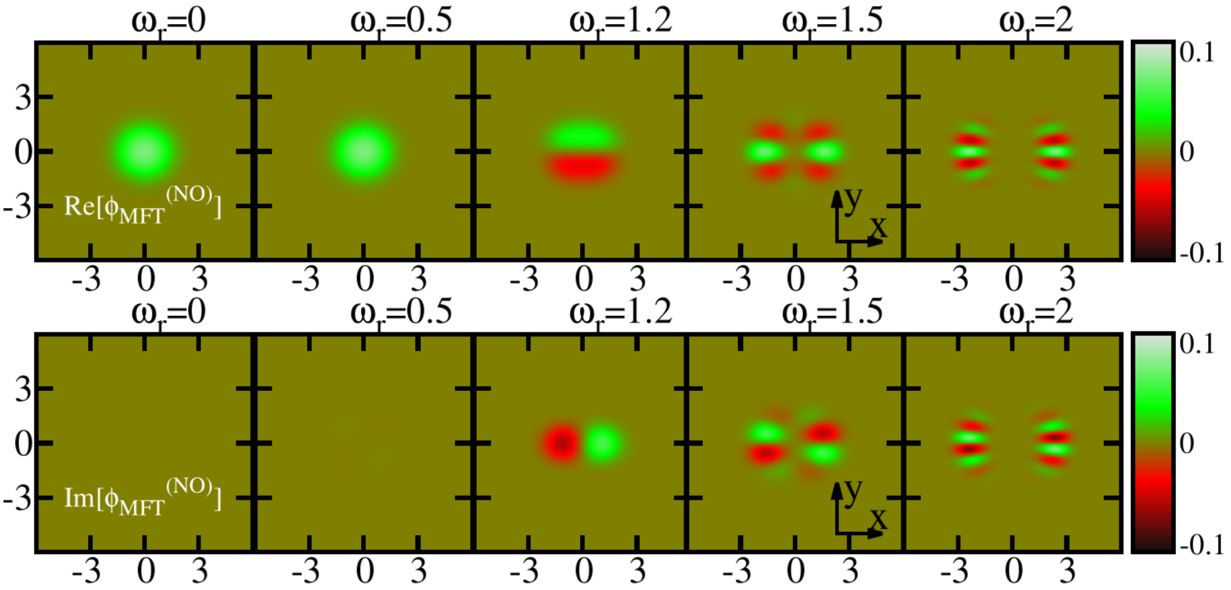

FIG. S8. Variation of the real and imaginary components of the mean-field natural orbital, ϕ_{MFT} with respect to different rotation frequencies, ω_r for elongated trap.

faster rotations, $\omega_r = 1.5$, ϕ_1 and ϕ_2 show two separated nodes. The similar behavior of both ϕ_1 and ϕ_2 compliment the two-fold fragmentation appeared for faster rotations.

Figure (S8) displays the real and imaginary components of the single mean-field orbital.

S6. ZOOMING IN THE RESONANCE REGIME

This section explains the various features emerged in the resonance regime, both in the mean-field as well as the many-body properties for the elongated trap. The distinguishable features around the resonance regime are as in the following,

(1) The resonance behaviour is evident both in the mean-field and many-body properties.

(2) The mean-field resonance has a narrow peak, however the many-body resonance has a wider width.

(3) The mean-field resonance starts slightly earlier than the many-body resonance.

(4) At the resonance frequency, the condensate absorbs significant angular momentum.

(5) The mean-field and many-body resonances can be appeared either in the same or in the opposite directions for quantum fluctuations.

To understand these distinct resonance features deeper, we analyze the behaviour of the one-body density per particle in the resonance regime computed both at the mean-field

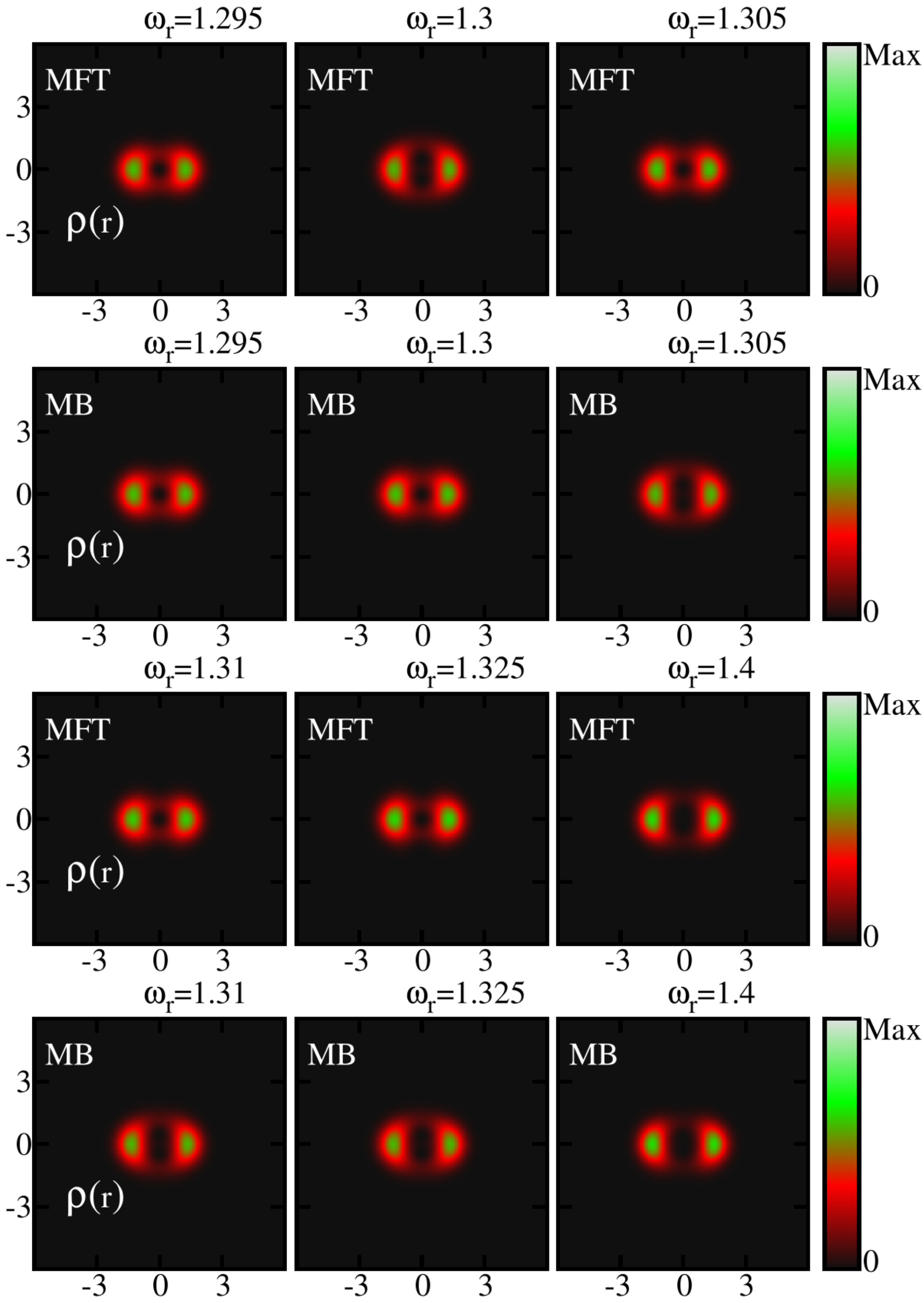

FIG. S9. The one-body densities per particle in the resonance regime computed at the mean-field, MFT and many-body, MB levels for elongated trap.

and many-body levels, see Figure (S9). The densities are shown for six specific rotation frequencies, ω_r. It is observed that the blowing of the density emerged at the resonance regime; for mean-field density at $\omega_r = 1.3$ and for many-body density at $\omega_r = 1.305$. This indicates the absorption of the angular momentum in the condensate. The mean-field density returns back to the non-resonant shape at $\omega_r = 1.305$. This explains the narrow peak appeared in the mean-field properties [see Fig.(3),(4) and (5) in the main text]. However,

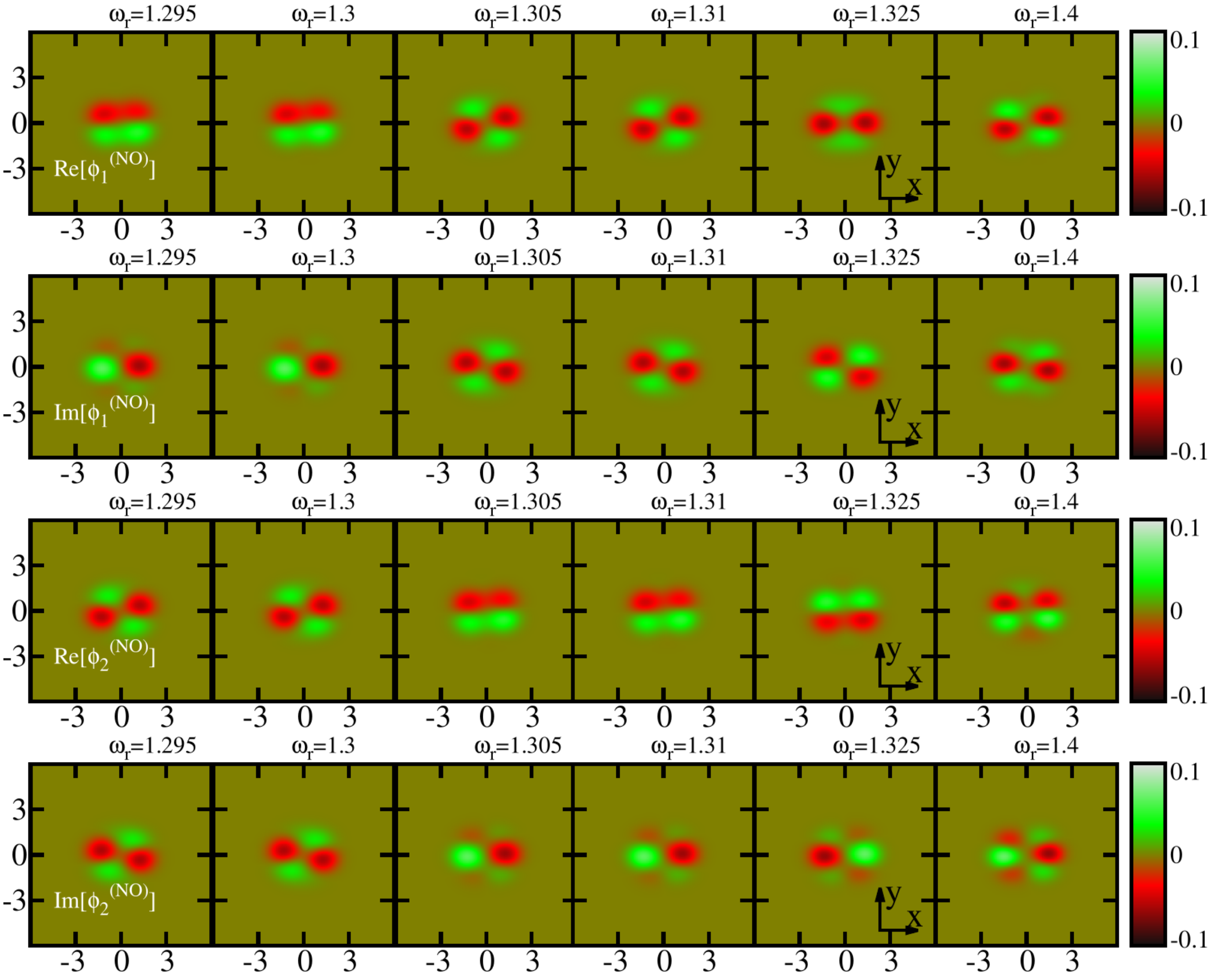

FIG. S10. Variation of the real and imaginary components of the two leading natural orbitals, ϕ_1, ϕ_2 in the resonance regime for elongated trap.

the many-body density clarifies that the many-body resonance appears slightly later around frequency $\omega_r = 1.305$ and it sustains like that. This explains the observed width in the many-body resonance, [see Fig. (3), (4) and (5)].

We further investigate the many-body and mean-field natural orbitals to get a more clear picture of the resonance condition. Figure (S10) presents the real and imaginary

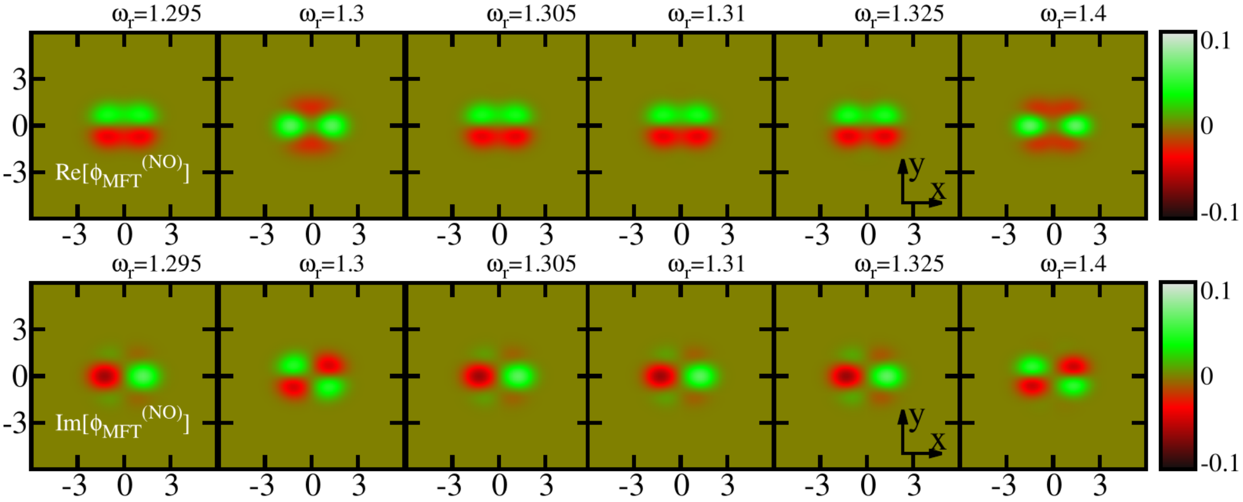

FIG. S11. Variation of the real and imaginary components of the mean-field natural orbital, ϕ_{MFT} in the resonance regime for elongated trap.

components of the two leading many-body natural orbitals, ϕ_1 and ϕ_2 in the resonance regime. It is observed that orbital nodes are odd before the resonance frequency. At the resonance frequency, $\omega_r = 1.305$, the ϕ_1 node becomes even and sustains this pattern. Similar behaviour is observed in the case of ϕ_2. Before resonance it remains even and then becomes odd at the resonance frequency. The rotational symmetry of the system changes in the resonance regime. Hence, the many-body natural orbitals are sensitive to the resonance condition. Similarly, the mean-field natural orbital is also very responsive to the resonance condition as evident from Figure (S11) [mean-field resonance starts at $\omega_r = 1.3$].

[1] A. U. J. Lode, K. Sakmann, O. E. Alon, L. S. Cederbaum, and A. I. Streltsov, Phys. Rev. A **86**, 063606 (2012).

[2] A. U. J. Lode, *Tunneling Dynamics in Open Ultracold Bosonic Systems: Numerically Exact Dynamics–Analytical Models–Control Schemes* (Springer, 2014).

[3] A. U. J. Lode, C. Lévêque, L. B. Madsen, A. I. Streltsov, and O. E. Alon, Rev. Mod. Phys. **92**, 011001 (2020).

[4] L. Cohen and C. Lee, J. Math. Phys. **26**, 3105 (1985).

[5] O. E. Alon, Symmetry **11**, 1344 (2019).

[6] O. E. Alon, J. Phys.: Conf. Ser. **1206**, 012009 (2019).
